# Altered T-Cell Responses by the Periodontal Pathogen *Porphyromonas gingivalis*

**DOI:** 10.1371/journal.pone.0045192

**Published:** 2012-09-12

**Authors:** Hazem Khalaf, Torbjörn Bengtsson

**Affiliations:** Division of Clinical Medicine, School of Health and Medical Sciences, Örebro University, Örebro, Sweden; International Center for Genetic Engineering and Biotechnology, India

## Abstract

Several studies support an association between the chronic inflammatory diseases periodontitis and atherosclerosis with a crucial role for the periodontal pathogen *Porphyromonas gingivalis*. However, the interplay between this pathogen and the adaptive immune system, including T-cells, is sparsely investigated. Here we used Jurkat T-cells to determine the effects of *P. gingivalis* on T-cell-mediated adaptive immune responses. We show that viable *P. gingivalis* targets IL-2 expression at the protein level. Initial cellular events, including ROS production and [Ca^2+^]_i_, were elevated in response to *P. gingivalis*, but AP-1 and NF-κB activity dropped below basal levels and T-cells were unable to sustain stable IL-2 accumulation. IL-2 was partially restored by Leupeptin, but not by Cathepsin B Inhibitor, indicating an involvement of Rgp proteinases in the suppression of IL-2 accumulation. This was further confirmed by purified Rgp that caused a dose-dependent decrease in IL-2 levels. These results provide new insights of how this periodontal pathogen evades the host adaptive immune system by inhibiting IL-2 accumulation and thus attenuating T-cell proliferation and cellular communication.

## Introduction

Accumulating amount of data during recent years support an association between periodontitis and atherosclerosis, which both are inflammatory conditions involving different kinds of immune cells [1]. The role of T-cells in atherosclerosis is well established [2], [3], however the involvement of T-cells in the pathogenesis and progression of periodontal disease is not fully elucidated [4]. T-cell activation and subsequent IL-2 secretion, which acts to further promote T-cell proliferation, play an important immune regulatory role. Disruption of IL-2 transcription and expression leads to T-cell anergy [5] and as a consequence this would result in an alteration of the antibody-based immunity by B-cells [6]. Andrukhov and colleagues [7] reported a significant decrease in serum IL-2 levels in patients suffering from periodontitis compared to healthy controls. Furthermore, regulatory T-cells and their release of the anti-inflammatory cytokine IL-10 have, in response to IL-2, been shown to exert anti-atherogenic effects [3], [8], [9]. Both CD4^+^ and CD8^+^ T-cells are present in atherosclerotic lesions [10], and may upon activation amplify the inflammatory condition in the atherosclerotic plaque through secretion of cytokines. Sasaki and colleagues [11] suggested the use of anti-CD3 to prevent atherosclerosis development and progression. They showed that administration of anti-CD3 activated regulatory T-cells and reduced atherosclerotic lesions and accumulation of other immune cells. Unravelling the effects of the periodontal pathogen *Porphyromonas gingivalis* on T-cells will contribute to the clarification of the mechanisms applied by this pathogen to evade host immune responses and cause disease.

*P. gingivalis* is an anaerobic, gram-negative rod associated with periodontal disease progression including bone and tissue destruction [12]. Lamont and colleagues [13] showed that *P. gingivalis* could invade and translocate into the cytosol within gingival epithelial cells, demonstrating a possible mechanism for its establishment, replication and subsequent pathogenesis by evading the host immune system. Similar results were observed in heart and aortic endothelial cells [14], indicating an association between *P. gingivalis*-dependent periodontitis and cardiovascular disease. Another mechanism used by *P. gingivalis* to evade the immune system is through its ability to inhibit CXCL-8 expression [15], and as a consequence impair immune cell recruitment.

Several factors contribute to the pathogenesis of *P. gingivalis*, including LPS and cysteine proteinases. Establishment and growth of *P. gingivalis* has been associated with its production and secretion of proteinases. These enzymes are divided into arginine-specific (Rgp) and lysine-specific (Kgp) gingipains [16]. RgpA-Kgp complexes have been reported to inactivate the T-lymphocyte-derived cytokines IL-4 and IL-5 [17] that are important for the activation and proliferation of B-lymphocytes. Even though cytokines and chemokines are expressed in response to *P. gingivalis*, their release and subsequent action on leukocyte migration is thus modulated due to the enzymatic activity of proteinases that cleave and inhibit the biological properties of different cytokines, including CXCL-8 [18] and TNF [19]. It is therefore important to determine the interactions between *P. gingivalis* and different host immune cells, as well as possible alterations in inflammatory gene regulation.

It is important to analyse T-cell responses to *P. gingivalis* infection, since this periodontal pathogen has been shown to be translocated with T-cells in atherosclerotic plaques. We hypothesize that *P. gingivalis* is able to suppress T-cell-derived responses, which benefits the pathogen to establish itself and proliferate. The aim of the present study was to characterize the effects of *P. gingivalis* on T-cell-mediated inflammatory responses and gene regulation.

## Materials and Methods

### Cell culture conditions

Jurkat T-cells cells (E6-1, ATCC) were maintained in 90% RPMI 1640 medium (Fisher scientific, Austria) with 1.5 mM L-glutamine (Invitrogen, USA) and supplemented with 10% fetal bovine serum (Invitrogen). The cells were incubated in a stable environment at 95% air, 5% CO_2_ and 37°C.

### Bacterial culture conditions and preparation

*Porphyromonas gingivalis* ATCC 33277 (American Type Culture Collection, Manassas, VA) was grown under anaerobic conditions (80% N_2_, 10% CO_2_, and 10% H_2_) at 37°C in an anaerobic chamber (Concept 400 Anaerobic Workstation; Ruskinn Technology Ltd., Leeds, United Kingdom). The bacteria were cultured for 3 days in fastidious anaerobe broth (29.7 g/liter, pH 7.2) before being washed and resuspended in Krebs-Ringer glucose buffer (KRG) (120 mM NaCl, 4.9 mM KCl, 1.2 mM MgSO_4_, 1.7 mM KH_2_PO_4_, 8.3 mM Na_2_HPO_4_, and 10 mM glucose, pH 7.3). The bacterial concentration was adjusted to correlate with approximately 10^9^ CFU/ml, which was determined by viable count where the bacteria were grown on fastidious anaerobe agar (46.0 g/liter supplemented with L-tryptophan 0.1 g/liter, pH 7.2; Lab M, Lancashire, United Kingdom) for 5 days.

Heat-killed *P. gingivalis* and heat-inactivated *P. gingivalis* supernatants were prepared following incubation at 70°C for 1 h. To ensure that the bacteria were killed, 10 µl of the heat-killed suspension was spread on a fastidious anaerobe agar plate and incubated at 37°C for 5 days. The absence of colony formation was used as an indicator that no viable bacteria were present in the suspension. *P. gingivalis* supernatants were sterile filtered through a 0.2 µm filter before being used. Both *P. gingivalis* and its supernatant were used fresh for every experiment.

Two selected inhibitors of cysteine proteinases (Leupeptin, Roche Diagnostics Corporation, USA and Cathepsin B Inhibitor II, Calbiochem, Germany) were used to determine the role of Arg- and Lys-gingipain activities. Viable *P. gingivalis* were incubated with different concentrations of the inhibitors for 1 h prior to stimulation of Jurkat T-cells. To further assess the contribution of gingipains, purified Arg-gingipain B (RgpB, Athens GA, USA) was used.

*E. coli* MG1655 were grown on Luria-Bertani (LB) plates and incubated at 37°C overnight. Single colony was inoculated into 10 ml LB and the tube was incubated at 37°C overnight on shaker set at 200 rpm. The bacteria were then harvested for 10 min at 3000×g, washed with 3 ml KRG and re-suspended in KRG.

### Isolation of primary cells

PBMC were isolated by the density gradient medium Ficoll-Paque™ Plus (Amersham Biosciences, Sweden) according to the manufacturers' instructions. Briefly, freshly collected blood from healthy donors was diluted with an equal volume of PBS, and 4 ml were carefully layered on top of 3 ml Ficoll-Paque Plus. The tubes were centrifuged at room temperature for 30 min at 300×g. PBMC were recovered from the interface and washed twice with PBS to remove excess Ficoll-Paque Plus and platelets. The cells were suspended in RPMI media supplemented with 10% foetal bovine serum and incubated in a stable environment at 95% air, 5% CO_2_ and 37°C for 2 days. Suspended cells were recovered, washed and cultured in a separate T75 flask for 24 h. The cells were then used to determine IL-2 expression in response to *P. gingivalis*.

### Enzyme-linked immunosorbent assay (ELISA)

ELISA was performed on supernatants from challenged Jurkat T-cells to quantify IL-2 (BD OptEIA Set Human IL-2, BD Biosciences, USA) according to the manufacturer's instructions. Briefly, Jurkat T-cells were either pre-treated with *P. gingivalis* or bacterial supernatant for 1 h followed by stimulation with 50 ng/ml PMA and 1 µg/ml Calcium Ionophore (Calcium Ionophore A23187 mixed calcium magnesium salt, Sigma #C5149, USA) or stimulated with PMA and Calcium Ionophore prior to treatment with *P. gingivalis* or bacterial supernatant. The cells were thereafter centrifuged at 95×g for 5 min and the supernatants were collected and stored at −80°C until use.

### Transfection and luciferase measurement

Activator protein (AP)-1 and nuclear factor (NF)-κB activity were measured by using luciferase reporter plasmids. Briefly, reporter plasmid (pAP1-Luc, NF-κB-Luc), internal control plasmid (Renilla) (Promega, USA) and lipofectamine 2000 (Invitrogen, USA) were added to each well at 0.54 µg/well, 0.06 µg/well and 1.5 µl/well, respectively. Initially, reporter plasmid and Renilla were mixed separately with OptiMEM (Gibco, USA). After 5 min of incubation at room temperature, lipofectamine 2000 was added and the mixture was incubated further for 20 min at room temperature. The transfection was allowed to proceed overnight at 37°C, after which, the cells were centrifuged, the media removed and fresh pre-warmed media added. The cells were lysed and luciferase activity was measured using the Dual-Luciferase® reporter assay system (Promega, USA) according to the manufacturer's instructions on a TD 20/20 luminometer (Turner Designs, Sunnyvale, CA).

### Measurement of ROS production

ROS production in Jurkat T-cells was analyzed using a lumiaggregometer (Chrono-Log Corp., Havertown, PA). Briefly, Jurkat T-cells (10^6^ cells/ml) were suspended in complete RPMI 1640 media supplemented with 10% FBS containing 50 µM luminol and 4 U/ml HRP. The cells were incubated at 37°C for 15 min, at 800 rpm, before being stimulated with 10^8^ CFU/ml *P. gingivalis* for 30 min, during which time chemiluminescence was registered.

### Measurement of [Ca^2+^]_i_

Cytosolic Ca^2+^ concentration was measured by using the fluorescent indicator Fura-2. Briefly, Jurkat T-cells were washed twice and resuspended in KRG to yield a cell-density of 10^6^ cells/ml. The cells were loaded with 4 µM Fura-2-acetoxymethylester (AM) for 40 min during gentle agitation. The cells were then washed twice. The extracellular Ca^2+^ concentration was set to 1 mM by addition of CaCl_2_ and intracellular Ca^2+^ concentration was determined in 2 ml aliquots at 37°C, 300 rpm using a Hitachi F2000 spectrofluorometer (Hitachi Ltd. Tokyo, Japan). Fluorescence emission and excitation was registered at 510 nm and 340/380 nm, respectively. Maximal and minimal ratios were determined following addition of 0.1% Triton X-100 and 24 mM EGTA. The change in intracellular Ca^2+^ concentration was calculated by using the equation described by Grynkiewicz and colleagues [20]. Calcium Ionophore was used as a positive control.

### Reverse transcription quantitative PCR (RT-qPCR)

RT-qPCR was used to determine gene expression levels of *il-2* in response to viable and heat-killed *P. gingivalis*. Briefly, Jurkat T-cells were pre-treated with *P. gingivalis* for 1 h, followed by stimulation with PMA/Ionophore for 24 h. RNA was extracted using RNeasy® Plus Micro Kit (Qiagen, USA) according to the manufacturer's recommendations. Reverse transcription was performed using Maxima® First Strand cDNA Synthesis Kit (Fermentas, Sweden). The following primer sequences were used: forward- ACCTCAACTCCTGCCACAATGTAC, reverse- TCAGTTCTGTGGCCTTCTTGGGCA. Thermal cycling conditions for SYBR Green (Fermentas) consisted of a denaturation step at 95°C for 10 min followed by 40 cycles of 95°C for 15 s and 60°C for 60 s. Gene expression was analyzed using a 7900 HT real-time PCR instrument (Applied Biosystems). The obtained Ct values were normalized against GAPDH. Relative quantification of gene-expression was determined by using the ΔΔCt method. The ΔCt was calculated by subtracting the Ct of GAPDH from the Ct of *il-2* for each sample. The ΔΔCt was calculated by subtracting the ΔCt of the control sample from the ΔCt of each treated sample. Fold change was generated by using the equation 2^−ΔΔCt^.

### Fluorescence microscopy

Jurkat T-cells were stimulated with FITC-labeled *P. gingivalis* (10^8^ CFU/ml, MOI:100) for 24 h and fixation overnight in 4% paraformaldehyde (PFA) at 4°C. F-actin was visualized by incubating the cells with 2 units Alexa Fluor® 594 phalloidin (Invitrogen) and 100 µg/ml lysophosphatidylcholine in darkness for 1 h at room temperature. The cells were washed and mounted on a coverslip. Adherence of *P. gingivalis* to Jurkat T-cells was analyzed by confocal microscopy (Leica Microsystems, Heidelberg, Germany).

### Statistical analysis

Statistical significant differences were determined using two-tailed Student's *t*-test (*p<0.05; **p<0.01; ***p<0.001).

## Results

### P. gingivalis triggers [Ca^2+^]_i_ changes

**Figure 1:**
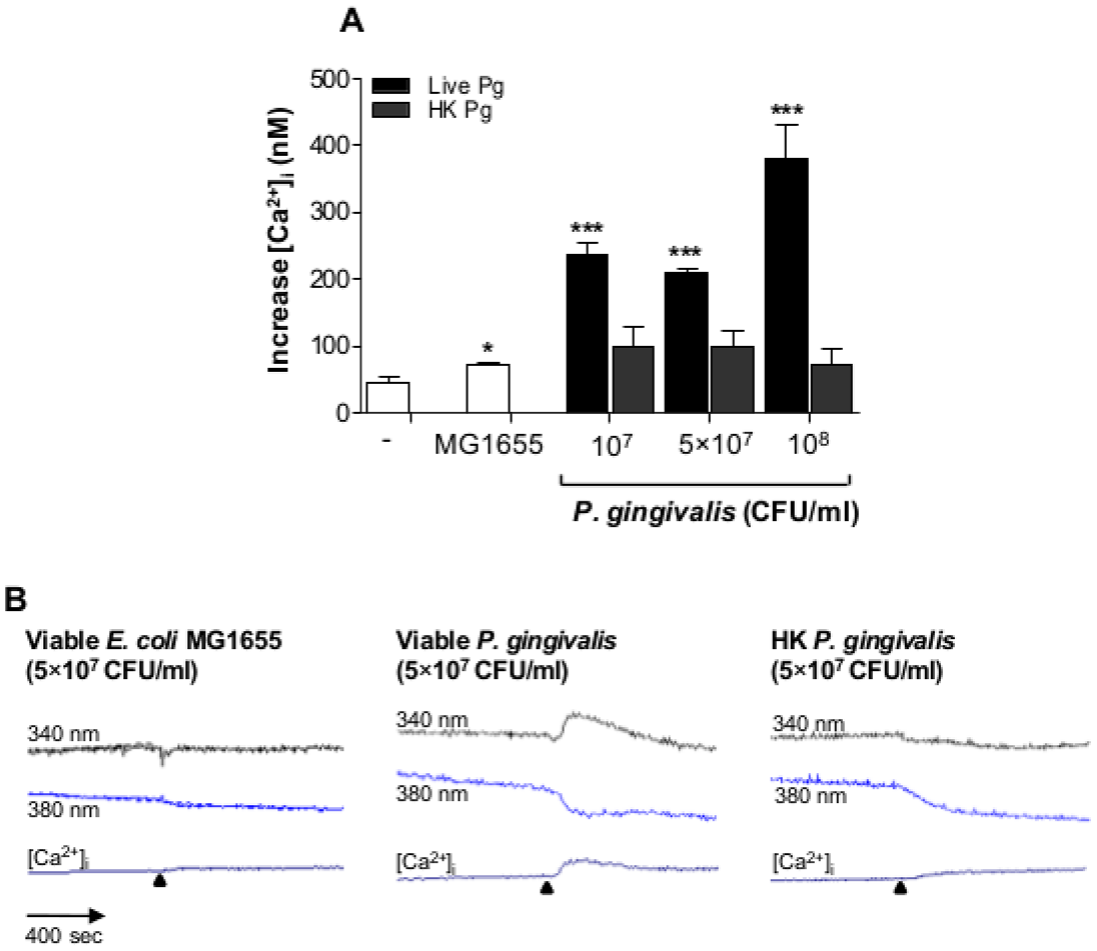
Viable *P. gingivalis* increased [Ca^2+^]_i_ concentration in Jurkat T-cells. [Ca^2+^]_i_ was measured by loading Jurkat T-cells (10^6^ cells/ml) with the fluorescent indicator Fura-2. **A**- Cells were exposed to the indicated concentrations of either viable (black bars)- or heat-killed (HK, grey bars) *P. gingivalis* or Viable *E. coli* MG1655 (5×10^7^ CFU/ml, MOI:50). Ca^2+^ release was induced in response to viable, but not heat-killed bacteria (MOI:10, 50 and 100, respectively). **B**- Representative curves of the excitation wavelengths 340 nm and 380 nm as well as the calculated Ca^2+^ concentrations following treatment of Jurkat T-cells with viable *E. coli* MG1655, viable *P. gingivalis* and heat-killed *P. gingivalis*, respectively. The arrows indicate the starting point of stimulation. Data shown are the mean±SD of three independent experiments. *-p<0.05; ***-p<0.001 (Student's *t*-test).

Changes in intracellular free calcium concentration [Ca^2+^]_i_ is an important action for the establishment of a proper response to foreign pathogens. This prompted us to determine the levels of [Ca^2+^]_i_ in Jurkat T-cells in response to viable- and heat-killed *P. gingivalis*. The basal [Ca^2+^]_i_ levels of unstimulated cells was around 45±14 nM. Viable *P. gingivalis* increased [Ca^2+^]_i_ by 5.1, 4.5 and 8.3 fold in response to 10^7^, 5×10^7^ and 10^8^ CFU/ml, respectively (figure 1a). However, T-cells did not respond when *P. gingivalis* were heat-killed (HK, figure 1a). Representative traces of the calcium changes following treatment of Jurkat T-cells with viable *E. coli* MG1655, viable *P. gingivalis* and heat-killed *P. gingivalis* are shown in figure 1b. As a positive control we used the calcium ionophore ionomycin, which caused a significant increase in [Ca^2+^]_i_ by ∼78 fold (data not shown). Viable *E. coli* MG1655 (5×10^7^ CFU/ml) was used as a control, and resulted in minor changes in [Ca^2+^]_i_.

### P. gingivalis binds to T-cells and induces ROS production

**Figure 2:**
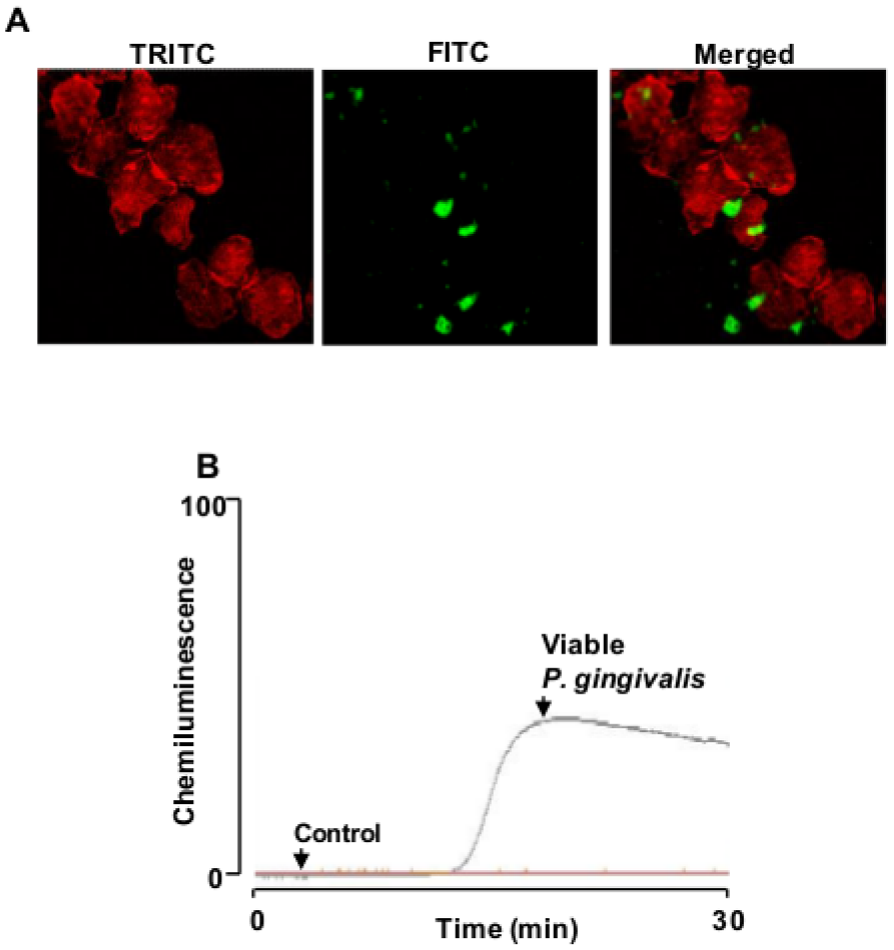
*P. gingivalis* is able to attach and induce ROS production in Jurkat T-cells. **A**- Jurkat T-cells cells were stimulated with FITC-labeled *P. gingivalis* (10^8^ CFU/ml, MOI:100) for 24 h and analyzed by confocal microscopy. Magnification is ×63, with a 2× digital zoom. **B**- ROS production in Jurkat T-cells was detected by luminol-amplified chemiluminescence following treatment with 10^8^ CFU/ml of viable *P. gingivalis* (MOI:100) for 30 min. Shown is a representative graph of five independent experiments.

The ability of *P. gingivalis* to attach to Jurkat T-cells and cause cell aggregation was shown by labeling the bacteria with FITC and stain the actin cytoskeleton with Alexa Fluor 594 phalloidin (figure 2a). We found that *P. gingivalis* efficiently binds to Jurkat T-cells and stimulate morphological changes and aggregate formation. As a consequence, bactericidal molecules, such as reactive oxygen species (ROS) may be released of *P. gingivalis*- T-cells interaction. ROS may function either, at low concentrations, as signaling molecules derived from different metabolic processes or, at high concentrations, as bactericidal toxic molecules produced by NADPH-oxidases. By using luminol-dependent chemiluminescence, changes in ROS production over time was analyzed in Jurkat T-cells following exposure to *P. gingivalis*. The cells where either left untreated, or exposed to viable *P. gingivalis* for 30 min. After a lag phase of around 12–14 min, viable *P. gingivalis* caused an extensive and long-lasting ROS production reaching maximum after 20 min (figure 2b).

### Viable P. gingivalis inhibits IL-2 expression and accumulation

**Figure 3:**
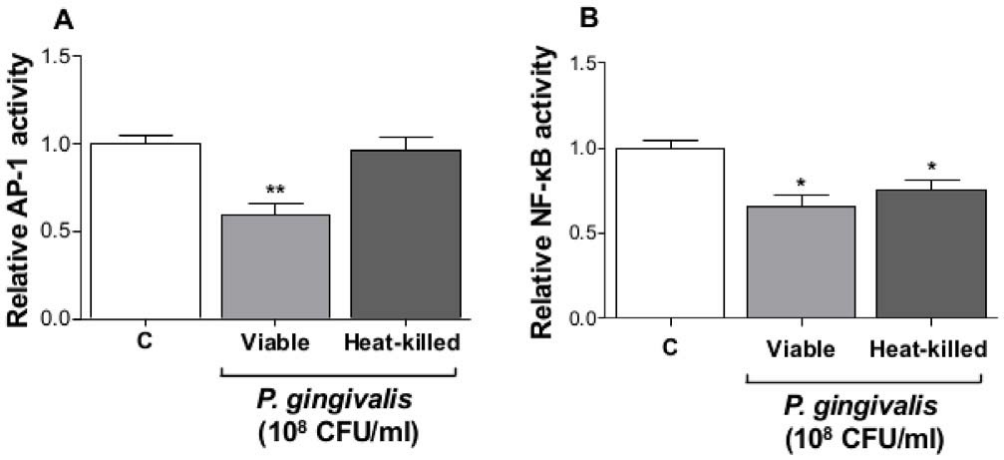
*P. gingivalis* suppressed AP-1 and NF-κB activity. Jurkat T-cells (10^6^ cells/ml) were transfected with either AP-1 (**A**) or NF-κB (**B**) luciferase reporter plasmids. The cells were treated with viable or heat-killed *P. gingivalis* (10^8^ CFU/ml, MOI:100) for 24 h. AP-1 and NF-κB activation were determined by measuring luciferase activity, which was normalized against the internal control *Renilla*. *-p<0.05; **-p<0.01 (Student's *t*-test).

**Figure 4:**
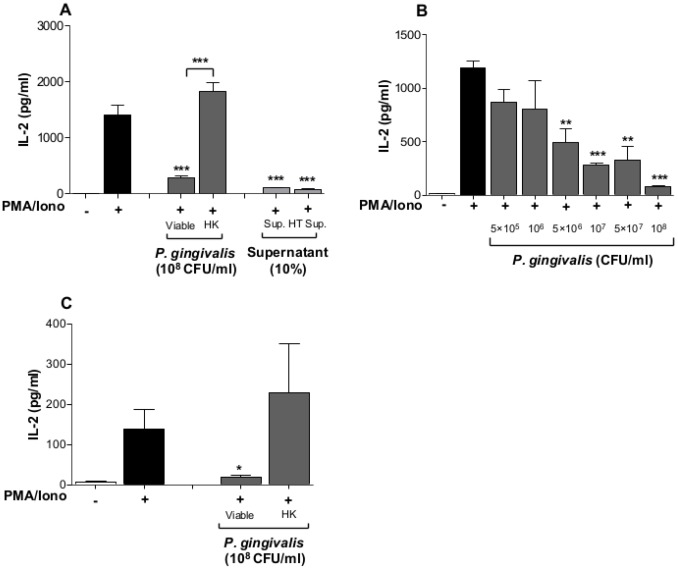
IL-2 accumulation decreases in response to viable *P. gingivalis* and its derived supernatant. **A-** Jurkat T-cells (10^6^ cells/ml) were pre-treated with 10^8^ CFU/ml of viable or heat-killed (HK) *P. gingivalis* (MOI:100) as well as 10% untreated- or heat-treated (HT) supernatant from *P. gingivalis* broth cultures for 1 h. The cells were then stimulated with 50 ng/ml PMA and 1 µg/ml Calcium ionophore for 24 h. IL-2 accumulation was significantly reduced by viable, but not heat-killed *P. gingivalis* in Jurkat T-cells, while both untreated and heat-treated bacterial supernatant resulted in a significant IL-2 reduction. **B-** T-cells were pre-treated with the indicated concentrations of viable *P. gingivalis* (MOI:0.5, 1, 5, 10, 50 and 100, respectively) for 1 h followed by stimulation with 50 ng/ml PMA and 1 µg/ml Calcium ionophore for 24 h. IL-2 accumulation was reduced in a dose-dependent manner. **C-** Primary cells were isolated as described in materials and methods. Cells were pre-treated with viable or heat-killed *P. gingivalis* for 1 h, followed by stimulation with 50 ng/ml PMA and 1 µg/ml Calcium ionophore for 24 h. Viable, but not heat-killed *P. gingivalis* (MOI:100) resulted in a significant reduction in IL-2 accumulation. *-p<0.05; **-p<0.01; ***-p<0.001 (Statistical significance between different treatments and the positive control PMA/Iono, Student's *t*-test).

Alteration in the levels of intracellular calcium and ROS prompted us to determine AP-1 activity in response to *P. gingivalis*. Jurkat T-cells were transfected with luciferase-reporter plasmids containing cis-acting elements for AP-1 or NF-κB, followed by exposure to viable or heat-killed *P. gingivalis*. Viable, but not heat-killed, *P. gingivalis* caused a significant inhibition of AP-1 activity (figure 3a), while both viable and heat-killed *P. gingivalis* reduced basal level of NF-κB activity (figure 3b). Considering the apparent effects on Jurkat T-cell signaling involving calcium, ROS and the transcription factors AP-1 and NF-κB, we then determined whether *P. gingivalis* and its supernatant affected accumulation of IL-2. The PMA/Ionophore-induced IL-2 accumulation after 24 h was significantly decreased by pre-treatment with viable bacteria, while exposure to heat-killed bacteria resulted in increased IL-2 levels, compared to the positive control (figure 4a). Pre-exposure of Jurkat T-cells to either untreated or heat-treated *P. gingivalis* supernatant resulted in a significant reduction in IL-2 levels. A viability assay showed that the observed inhibition of IL-2 accumulation by viable *P. gingivalis* was not due to cell death. Cell-density increased by 20% after stimulation of Jurkat T-cells with viable *P. gingivalis* for 24 h, compared to 50% increase in the control and cells treated with heat-killed *P. gingivalis* (data not shown). Furthermore, treatment of Jurkat T-cells with either viable or heat-killed *P. gingivalis*, without PMA/Ionophore, did not alter IL-2 levels compared to the untreated negative control (data not shown). Inhibition of IL-2 accumulation by bacterial supernatants was shown to be due to small molecular weight compounds present in the fastidious anaerobe broth, as determined by molecular mass fractionation, rather than any bacterial-derived compound(s) (data not shown).

Furthermore, pre-incubation of Jurkat T-cells with different concentrations of viable *P. gingivalis* showed that inhibition of IL-2 accumulation was dose-dependent (figure 4b). A final concentration of 5×10^6^ CFU/ml (MOI:5) of viable bacteria was sufficient to significantly reduce IL-2 accumulation by ∼2.4 fold, compared to the positive control PMA/Ionophore, while the highest concentration 10^8^ CFU/ml reduced IL-2 accumulation by ∼14 fold. We thereafter aimed to confirm these results by using primary cells isolated from healthy volunteers. Viable, but not heat-killed *P. gingivalis* caused a significant reduction in IL-2 accumulation (figure 4c).

### Viable P. gingivalis targets IL-2 at the protein level

**Figure 5:**
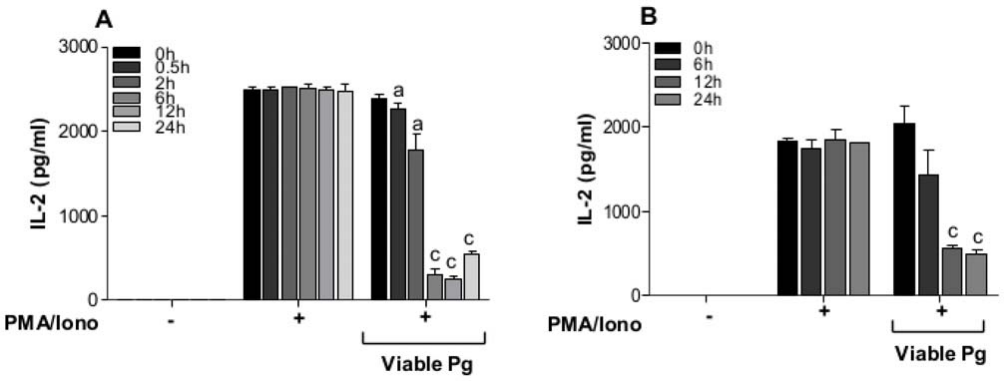
Viable *P. gingivalis* cleaves and prevents IL-2 accumulation. **A**- Jurkat T-cells (10^6^ cells/ml) were stimulated with 50 ng/ml PMA and 1 µg/ml Calcium ionophore for 24 h followed by exposure to viable *P. gingivalis* (Viable Pg, 10^8^ CFU/ml, MOI:100) for the indicated times. IL-2 accumulation was significantly decreased by *P. gingivalis* over time. **B**- Jurkat T-cells (10^6^ cells/ml) were stimulated with 50 ng/ml PMA and 1 µg/ml Calcium ionophore for 24 h. The cells were then removed and viable *P. gingivalis* (Viable Pg, 10^8^ CFU/ml, MOI:100) were added to cell-culture supernatants, containing secreted IL-2, for the indicated times. *P. gingivalis* is involved in cleaving and de-activating IL-2 proteins. The letters indicate significant differences compared to their respective positive control PMA/Iono at each specific time point. a-p<0.05; c-p<0.001 (Student's *t*-test).

In the experiments so far, the cells have been pre-exposed with either viable or heat-killed *P. gingivalis* to determine whether this treatment can alter intracellular mechanisms involved in IL-2 transcription and expression. In order to determine whether viable *P. gingivalis* affect produced and accumulated IL-2, Jurkat T-cells were first stimulated with PMA/Ionophore for 24 h, and then exposed to viable *P. gingivalis* for the indicated times (figure 5). IL-2 levels remained constant over time (0.5–24 h) in the untreated and the PMA/Ionophore-stimulated groups, while treatment with viable *P. gingivalis* resulted in a significant reduction in IL-2 levels already after 30 min and the levels continued to decrease over time (figure 5a). A second set of experiment was performed to determine whether the bacteria can alter the expression and subsequent release of IL-2. Jurkat T-cells were stimulated for IL-2 production as mentioned above, followed by centrifugation to exclude the cells. *P. gingivalis* was then added to the cell-free, IL-2 containing media for the indicated times (figure 5b). A similar trend was observed where *P. gingivalis* caused a significant reduction in IL-2 levels.

**Figure 6:**
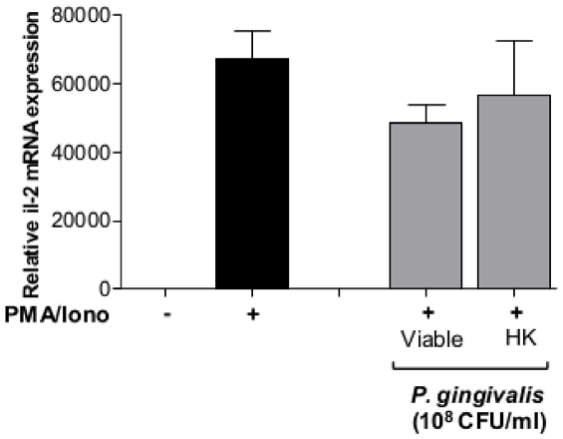
RT-qPCR analysis of *il-2* gene-expression in response to *P. gingivalis*. Jurkat T-cells (10^6^ cells/ml) were pre-treated with 10^8^ CFU/ml viable- or heat-killed (HK) *P. gingivalis* (MOI:100) for 1 h followed by stimulation with 50 ng/ml PMA and 1 µg/ml Calcium ionophore for 24 h. *Il-2* mRNA levels were not affected by viable or heat-killed *P. gingivalis*. ***-p<0.001 (Statistical significance between different treatments and the positive control PMA/Iono, Student's *t*-test).

We then aimed to determine whether the observed inhibition of the extracellular IL-2 accumulation was affected at the transcript level. Jurkat T-cells were pre-treated with viable- or heat-killed *P. gingivalis*, followed by induction with PMA/Ionophore. RT-qPCR analysis showed that *il-2* mRNA levels, induced by PMA/Ionophore, remained elevated in response to both viable- and heat-killed *P. gingivalis* treatment (figure 6).

### Degradation of IL-2 by arginine gingipains

**Figure 7:**
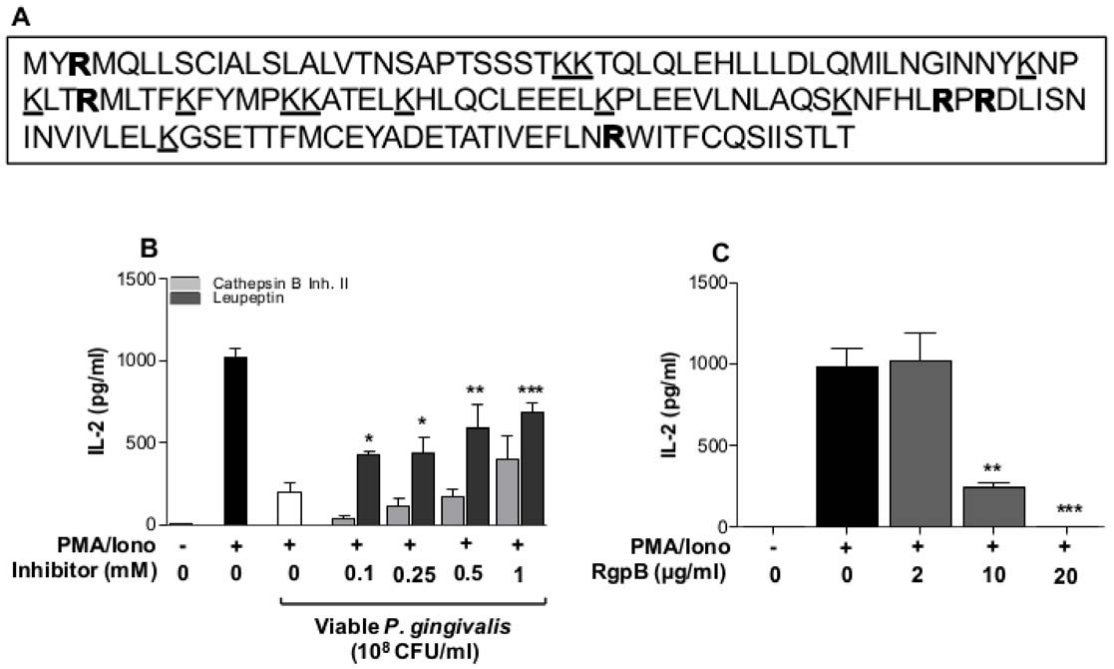
The proteinase inhibitor Leupeptin partially restored IL-2 accumulation. **A**- IL-2 amino acid sequence with predicted Rgp (bold)- and Kgp (underlined) cleavage sites. Five Rgp sites and 11 Kgp sites were found, source: http://www.ncbi.nlm.nih.gov/CCDS/CcdsBrowse.cgi?REQUEST=CCDS&DATA=CCDS3726. **B**- The involvement of Rgp and Kgp in IL-2 cleavage was determined by using Cathepsin B inhibitor II and Leupeptin. Viable *P. gingivalis* were incubated with the indicated concentration for 1 h prior to exposure of cells. Jurkat T-cells (10^6^ cells/ml) were pre-treated with 10^8^ CFU/ml of viable *P. gingivalis* (MOI:100) for 1 h followed by stimulated with 50 ng/ml PMA and 1 µg/ml Calcium ionophore for 24 h. IL-2 accumulation was partially restored by Leupeptin, but not by Cathepsin B inhibitor. The asterisks indicate significant differences compared to their respective control (Viable Pg, without Leupeptin). **C**- Purified RgpB resulted in a dose-dependent inhibition of IL-2 accumulation. The asterisks indicate significant differences compared to the positive control PMA/Iono. *-p<0.05; **-p<0.01 (Student's *t*-test).

The ability of viable *P. gingivalis* to cleave or in-activate the pre-accumulated IL-2 led us hypothesize that *P. gingivalis*-derived proteinases are involved in the inhibition of IL-2 accumulation. Analysis of the IL-2 amino acid sequence revealed 5 arginine cleavage sites and 11 lysine cleavage sites (figure 7a). In order to determine the involvement of these specific proteinases, viable bacteria were incubated with the selected arginine-specific and lysine-specific proteinase inhibitors Leupeptin and Cathepsin B inhibitor II, respectively, prior to exposure of Jurkat T-cells. The results showed that inhibition of IL-2 accumulation was partially dependent on the action of Rgp proteinases, either released and free in suspension or bound to the bacteria (figure 7b). Furthermore, treatment of PMA/Ionophore stimulated Jurkat T-cells with purified RgpB resulted in a dose-dependent inhibition of IL-2 accumulation (figure 7c).

## Discussion

T-cells are found in atherosclerotic plaques and their contribution to the progression of this inflammatory condition is well established [2]. Proliferation of effector T-cells in plaques requires IL-2, and the activity of effector T-cells is strictly controlled by regulatory T-cells [3]. Intracellular free Ca^2+^ is an important signaling molecule that is required for the activation of T-cells upon recognition of a foreign antigen [21]. Activation of the transcription factors AP-1, NFAT and NF-κB, and subsequent gene-expression and cytokine release requires a sustained Ca^2+^ influx [22]. These transcription factors are activated through Ca^2+^-dependent signaling proteins, including PKC [23] and calmodulin/calcineurin complex [24]. We found that intracellular Ca^2+^ levels increased in response to viable, but not heat-killed, *P. gingivalis*. The fact that only viable bacteria were able to elevate [Ca^2+^]_i_ may be due to that they possess intact fimbriae, which has been reported as an important feature, enabling the bacteria to attach, invade and induce an inflammatory response [25]. Furthermore, heat-sensitive bacterial proteinases have been reported to cleave and thus activate proteinase-activated receptor-2 (PAR-2), which is expressed on T-cells [26], leading to an increase in [Ca^2+^]_i_ [27].

Intracellular ROS production is important for elimination of invading pathogens and has been reported to influence T-cell activation [28]. In this study, we found that *P. gingivalis* binds to and aggregate T-cells and induces an extensive ROS production, which may reflect an ability of this pathogen to recognize specific cell surface receptors and induce intracellular signaling cascades involving ROS. *P. gingivalis* has previously been shown to affect the activity and/or expression of several cell surface receptors that are expressed on a variety of cells, including T-cells. These receptors include proteinase-activated receptors that are activated by *P. gingivalis*- derived proteinases [29]. However, microarray analysis showed an inhibitory effect of TCR expression by this periodontal pathogen [4]. Furthermore, Kitamura and colleagues reported that *P. gingivalis* was able to cleave CD4 and CD8 and thus impair T-cell activation [30]. *P. gingivalis* probably utilizes such a strategy to evade the host immune system, which benefits its establishment and proliferation. Several studies have reported the ability of *P. gingivalis* to invade host cells, including gingival epithelial cells [13] and heart endothelial cells [14] and that expression of fimbriae enables pathogen attachment and invasion [31]. Other strategies utilized by this pathogen to evade the host immune system are by affecting cells of the innate immune system. These effects are mainly due to the action *P. gingivalis*-derived proteinases and include proteolysis of CD14 on monocytes [32] and C5a receptor on neutrophils [33]. This would, as a consequence, lead to reduced bacterial recognition by monocytes and neutrophil migration, respectively.

Furthermore, viable *P. gingivalis* reduced AP-1 and NF-κB activity below basal levels. Transcriptional regulators, including AP-1 and NF-κB, are important for inflammatory gene-expression, such as CXCL-8 and IL-6 [34]. In addition, AP-1 has been shown to be an important regulator of IL-2 expression, in cooperation with NFAT [35], through PKC [36]. Mutation of the NF-κB site did not affect IL-2 expression, while mutation of the AP-1 site or PKC depletion almost revoked IL-2 release. These observations indicate that the MAPK pathway and the transcription factor AP-1 play an important role in the induction of inflammatory responses in Jurkat T-cells. We therefore aimed to determine IL-2 expression in response to *P. gingivalis*. Presence of viable *P. gingivalis* significantly inhibited PMA/Ionophore-induced IL-2 accumulation in Jurkat T-cells suspension. This effect may be due to the action of *P. gingivalis*-derived proteinases that have been reported to regulate several cytokines and chemokines. Kobayashi-Sakamoto and colleagues [18] reported the involvement of proteinases in degradation of CXCL-8 and MCP-1 and the Th2 cytokine IL-4 and IL-5 have also been shown to be targets for degradation by *P. gingivalis* proteinases [17]. However, receptor activator of NF-κB ligand (RANKL) is induced in Jurkat T-cells by *P. gingivalis* secreted compounds [37], while *P. gingivalis* outer membrane proteins were shown to induce IL-17 rather than RANKL [38]. These observations indicate that different *P. gingivalis*-derived components can differentially regulate cytokine expression.

We thereafter aimed to determine whether addition of *P. gingivalis* could alter a pre-stimulated accumulation of IL-2 and observed that only viable bacteria were able to reduce IL-2 levels and did that in a time-dependent manner. IL-2 was shown to be targeted at the protein level, since the transcript levels were not affected by *P. gingivalis*. This indicates that *P. gingivalis* regulates T-cell activation and proliferation by targeting the accumulation of IL-2, considering the importance of this cytokine in *P. gingivalis*-mediated T-cell proliferation [39].

*P. gingivalis*-derived proteinases have been reported to inhibit cytokine and chemokine expression [40]. This inhibitory effect is dependent on the enzymatic activity of these proteinases that cleave and inactivate several inflammatory markers, including TNF [19] and IL-6 [41]. Yun and colleagues [42] showed that *P. gingivalis*-derived proteinases activated T-cells through protease-activated receptors, but were also able to degrade CD27, which is a TNF receptor family member, as well as its ligand CD70, present on B-cells. These observations indicate that cellular communication is interrupted by *P. gingivalis* and may result in an impaired host immune response and less efficient clearance of an infection. The involvement of proteinases in the inhibition of IL-2 accumulation in response to viable *P. gingivalis*, was shown by the partial restoration of IL-2 in the presence of Leupeptin. Furthermore, purified gingipains completely antagonized IL-2 accumulation. By considering the IL-2 amino acid sequence, the number of arginine and lysine cleavage sites were predicted and corresponded to 5 and 11, respectively. However, IL-2 was shown to be targeted by Rgp rather than Kgp proteinases. This is in accordance with the observations made by Yun and colleagues [42] showing that T-cell activation and subsequent cellular communication are interrupted by arginine-specific cysteine proteinases.

The importance of sustained IL-2 levels for growth of regulatory T-cells has previously been reported [43]. The effects of *P. gingivalis* and its proteinases on T-cells are evident. We show that IL-2 accumulation is targeted by *P. gingivalis* at the protein level, and partially through suppression of AP-1, unraveling a mechanism applied by *P. gingivalis* to benefit its establishment by altering adaptive immune responses. Hence, alteration of IL-2 levels benefits bacterial establishment and may also contribute to progression of the inflammatory state in atherosclerosis, considering that IL-2 play an important role in the clonal expansion of regulatory T-cells. Furthermore, effector T-cells are found in atherosclerotic plaques and are considered to contribute to the progression of the inflammatory process. Identification of immune-regulatory compounds from *P. gingivalis* and the effects of these compounds on different T-cell subsets may be crucial in the development of new strategies to restrict further progression of atherosclerotic plaque formation and development.
